# Femtosecond Laser-Induced Periodic Surface Structures on Fused Silica: The Impact of the Initial Substrate Temperature

**DOI:** 10.3390/ma11081340

**Published:** 2018-08-02

**Authors:** Stephan Gräf, Clemens Kunz, Sebastian Engel, Thibault J. -Y. Derrien, Frank A. Müller

**Affiliations:** 1 Otto Schott Institute of Materials Research (OSIM), Friedrich Schiller University Jena, Löbdergraben 32, 07743 Jena, Germany; clemens.kunz@uni-jena.de (C.K.); sebastian.engel@uni-jena.de (S.E.); frank.mueller@uni-jena.de (F.A.M.); 2 HiLASE Centre—Institute of Physics of the Czech Academy of Sciences, Za Radnicí 828, 25241 Dolní Břežany, Czech Republic; derrien@fzu.cz

**Keywords:** fs-laser, laser-induced periodic surface structures (LIPSS), fused silica, substrate temperature, supra-wavelength LIPSS, CO_2_ laser, hydrodynamic instabilities

## Abstract

The formation and properties of laser-induced periodic surface structures (LIPSS) were investigated upon fs-laser irradiation of fused silica at different initial substrate temperatures, *T*_S_. For substrate heating between room temperature, *T*_RT_, and *T*_S_ = 1200 °C, a continuous wave CO_2_ laser was used as the radiation source. The surface structures generated in the air environment at normal incidence with five successive fs-laser pulses (pulse duration, *τ* = 300 fs, laser wavelength, *λ* = 1025 nm, repetition frequency, *f*_rep_ = 1 kHz) were characterized by using optical microscopy, scanning electron microscopy, and 2D-Fourier transform analysis. The threshold fluence of fused silica was systematically investigated as a function of *T*_S_. It was shown that the threshold fluence for the formation of low-spatial frequency LIPSS (LSFL) decreases with increasing *T*_S_. The results reveal that the initial spatial period observed at *T*_RT_ is notably increased by increasing *T*_S_, finally leading to the formation of supra-wavelength LIPSS. The findings are discussed in the framework of the electromagnetic interference theory, supplemented with an analysis based on thermo-convective instability occurring in the laser-induced molten layer. Our findings provide qualitative insights into the formation mechanisms of LIPSS, which allow improvements of the control of nanostructure formation to be made for corresponding applications of dielectric materials in the future.

## 1. Introduction

Since their first description by Birnbaum in 1965 [1], laser-induced periodic surface structures (LIPSS) have rapidly gained increasing attention [2]. The formation of LIPSS has been demonstrated as a universal phenomenon, occurring on almost all types of materials when irradiated close to their ablation threshold [3]. Due to their spatial periodicity, LIPSS are adequate for a large variety of applications in fields, such as sub-wavelength optics, tribology, and biomaterials engineering [2,4]. Based on their spatial characteristics, LIPSS are classified into low-spatial frequency LIPSS (LSFL, with a period, *Λ*, close to the laser wavelength, *λ*) [3,5,6,7] and high-spatial frequency LIPSS (HSFL, with *Λ* < *λ*/2) [2]. On metals and semiconductors, LSFL are predominantly characterized by an orientation perpendicular to the electrical field (E-field) vector [3]. In the case of band gap materials, the absorption of the high intensive fs-laser radiation can lead to a highly excited electronic state, possibly enabling a transient metal-like behavior [8]. According to the electromagnetic scattering theory of Sipe and accounting for the laser-excitation of fused silica [9,10], LSFL are characterized by a period, *Λ*, either close to *λ* or close to *λ*/*n* (*n* is the refractive index of the material), and an alignment predominantly parallel to the E-field vector [3,10,11]. HSFL are predominantly observed for the irradiation with pulses in the ps- to fs-range mainly for below band-gap excitation of transparent materials [3,12].

For metals and semiconductors, it is well-accepted that the formation mechanisms of LSFL lies in a spatially modulated energy deposition pattern resulting from the interference of incident laser radiation with excited surface electromagnetic waves [9,13]. A complementary approach to explain LIPSS formation is given by self-organization of the irradiated material via laser-induced thermal instabilities, resulting in material redistribution [14,15]. At metal surfaces, Gurevich et al. investigated the role of two-temperature heating dynamics in the context of periodic structure formation exposed to single ultrashort laser pulses [16]. The origin of HSFL is still unclear and remains under debate. Possible explanations include non-linear materials transport [17], second-harmonic generation [18], chemical surface alterations (e.g., oxidation) [19], and transient propagation effects in the laser-excited material [20,21].

The formation of LIPSS on glasses was mainly studied for fused silica, considering a large variety of influencing parameters, including the wavelength, *λ*, the number of incident laser pulses, *N*, the laser peak fluence, *F*, and the beam polarization [10,11,22,23,24,25,26,27,28]. Based on a comparison of different glasses with similar optical properties, it was shown that LIPSS formation is strongly determined by the chemical composition of the glasses, in particular by the respective viscosity [11]. In this context, LIPSS with spatial periods exceeding the laser wavelength (“supra-wavelength” LIPSS) have been demonstrated on silicate glasses [11]. The substrate temperature, *T*_S_, represents another important parameter, as it determines the viscosity of the material as well as the interaction with laser radiation in terms of the temperature-dependent optical properties of the material. The latter are characterized by the optical refractive index, *n*, the extinction coefficient, *k*, and the material band gap, *E*_g_. The substrate temperature has been the subject of several investigations concerning the ablation process and the damage threshold of fused silica and other materials [29,30,31]. However, the influence of *T*_S_ on the evolution process of LIPSS and particularly on the correlated morphology (e.g., spatial period and orientation of the structures) has rarely been the focus of research activities so far [32]. Recently, Tsibidis et al. attempted a theoretical description of possible hydrodynamic effects to describe the formation and orientation of LIPSS on dielectrics [33]. However, the role of the initial substrate temperature increase was missing and the formed LIPSS pattern solely resulted from the energy deposited by the fs-laser pulses and heat accumulation effects.

In the present study, the influence of *T*_S_ on the formation and properties of LIPSS is experimentally investigated on fused silica. For this purpose, a continuous wave (cw) CO_2_ laser is utilized as the heating source to generate a well-defined radial temperature profile on the substrate surface. The experimental results are discussed within the framework of the electromagnetic interference theory, supplemented by an analysis based on a thermo-convective instability model. The study provides experimental and theoretical insights into the formation of LIPSS in a hydrodynamic regime on fused silica irradiated with fs-laser pulses.

## 2. Materials and Methods

**Figure 1:**
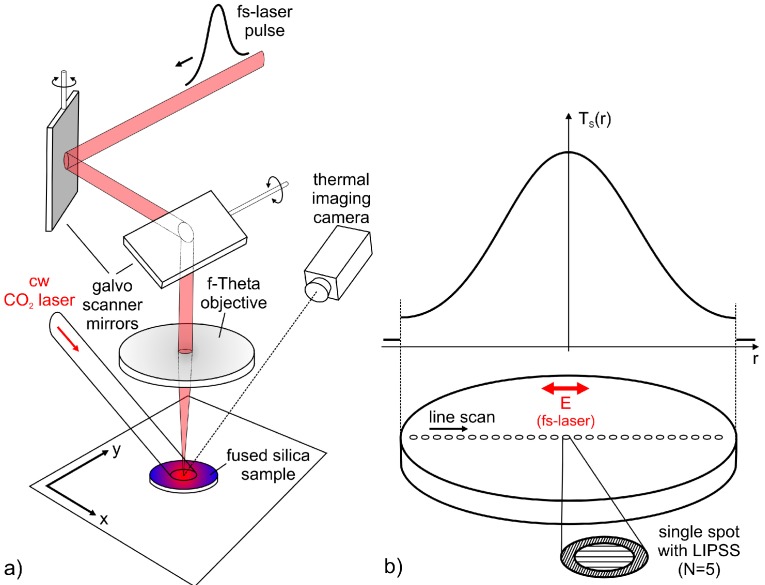
(**a**) Experimental setup and (**b**) generation of laser-induced periodic surface structures (LIPSS) at different substrate temperatures, *T*_S_(*r*), that depend on the radial position on the sample. Parameters of the fs-laser were *τ* = 300 fs (FWHM), *λ* = 1025 nm, *E*_imp_ ≤ 40 µJ, and *f*_rep_ = 1 kHz.

Figure 1a schematically illustrates the principle of the experimental setup. For the formation of LIPSS, a diode pumped Yb:KYW thin disc fs-laser system (JenLas D2.fs, Jenoptik, Jena, Germany) was used as the radiation source. The emitted linearly polarized laser radiation is characterized by a pulse duration, *τ* = 300 fs (FWHM), a central wavelength, *λ* = 1025 nm, pulse energies, *E*_imp_ ≤ 40 µJ, at a repetition frequency, *f*_rep_ = 1 kHz, and a Gaussian beam profile (*M*^2^ ~ 1.08). The pulsed laser beam was focused by using a galvanometer scanner (IntelliScan14, Scanlab, Puchheim, Germany), including a f-Theta objective (JENar, Jenoptik, Jena, Germany) with a focal length of *f*_L_ = 100 mm (*NA* ~ 0.05). Using the method proposed by Liu [34], the resulting focal spot diameter was measured to 2*w*_f_ = (24 ± 0.5) µm (1/*e*^2^ intensity). The uncertainty of the fs-laser peak fluence, *F* = 2*E*_imp_/(π·*w*_f_^2^), determined from the measured pulse energy, *E*_imp_, is estimated at <10%. A cw CO_2_ laser (SM1200P, FEHA, Halle, Germany) with a Gaussian intensity distribution (*λ* = 10.6 µm) and a beam diameter of about 5 mm was directed onto the sample surface to generate a radially symmetrical temperature profile, *T*_S_(*r*), at the sample surface. For this spot size, a power of 35 W was chosen to ensure sufficiently high temperatures and to prevent any alteration of the glass surface by the CO_2_ laser beam in terms of melting and vaporization. The resulting temperature profile was detected by using a thermal imaging camera (PI, optris, Berlin, Germany) with a temperature-range of up to 900 °C. An additional pyrometer (QKTRD 1085-1, Maurer, Kohlberg, Germany) was utilized to determine the maximum temperature at the center of the spot.

The fused silica samples (EN08, GVB, Herzogenrath, Germany) with a thickness of 0.7 mm and a diameter of 10 mm were ultrasonically cleaned in acetone and isopropanol. LIPSS were subsequently generated by irradiating the sample surface at normal incidence and at ambient air (Figure 1b). For this purpose, LIPSS were generated at different positions of the sample surface, each corresponding to a well-defined temperature, *T*_S_. Different values of *F* were chosen to investigate the influence of the fs-laser beam parameters on LIPSS formation. Every single spot was hit by *N* = 5 laser pulses and the direction of the linear polarization was adjusted, as indicated in Figure 1b.

Laser processed sample surfaces were ultrasonically cleaned in acetone and isopropanol, and subsequently characterized by scanning electron microscopy (SEM). For this purpose, the fused silica samples were sputtered with gold and examined in the SEM (EVO 40, Zeiss, Jena, Germany) at an accelerating voltage of 1–5 kV using a secondary electron detector. The spatial periods, *Λ*, of the LIPSS pattern were quantified by 2D-Fourier transform analysis (2D-FT) of the SEM micrographs. In this context, vertical bars displayed in the graphs indicate the width of the distribution of the corresponding 2D-FT operation. The size of the LIPSS pattern was evaluated by optical microscopy (OM). The utilized microscope (VHX-100K, Keyence, Osaka, Japan) was equipped with a wide-range zoom lens (VH-Z500, Keyence, Osaka, Japan).

## 3. Results and Discussion

### 3.1. Temperature Profile

**Figure 2:**
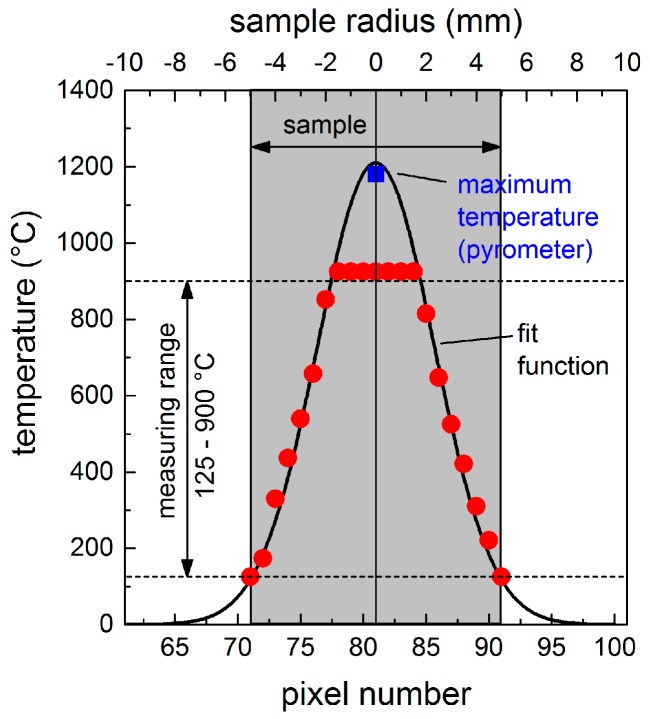
Experimentally measured local substrate temperatures (colored dots) and interpolated temperature profile *T*_S_(*r*) (black solid line) during cw CO_2_ laser irradiation.

In previous studies, CO_2_ lasers have demonstrated their suitability to heat glass samples due to the high absorption of glasses at wavelengths of *λ* > 5 µm [35]. For the fused silica used in the present study, the corresponding optical constants at *λ* = 10.6 µm and room temperature are *n* = 1.98, and *k* = 0.059, respectively. Thus, the reflectivity, *R*, is about 10% at normal incidence (*θ* = 0°), and can be reduced to *R* = 0 by using p-polarized radiation and the Brewster’s angle, *θ*_B_ = 63.2°, as the angle of incidence. Considering the optical penetration depth, *l*_α_ = 14 µm, this means that the CO_2_ laser radiation is completely absorbed by the material. After operating the CO_2_ laser for 20 s, a stationary temperature profile was established, representing the equilibrium between the absorbed energy and energy dissipated via heat conduction into the material and by thermal radiation from the surface. The corresponding temperature profile, *T*_S_(*r*), is illustrated in Figure 2. Due to the wide range of substrate temperatures, the experimental values with a maximum at the center and a minimum of about 130 °C at the edges of the sample were fitted with a Gaussian function. Using a pyrometer, a maximum temperature of about 1180 °C was detected in the center of the spot. This is in good agreement with the maximum temperature of 1200 °C predicted by the Gaussian fit function. Consequently, the determined temperature profile allows the direct relation between a certain position, *r*, at the sample surface and the corresponding local temperature, *T*_S_(*r*), during LIPSS formation.

### 3.2. LIPSS Formation in Dependence of Initial Substrate Temperature

**Figure 3:**
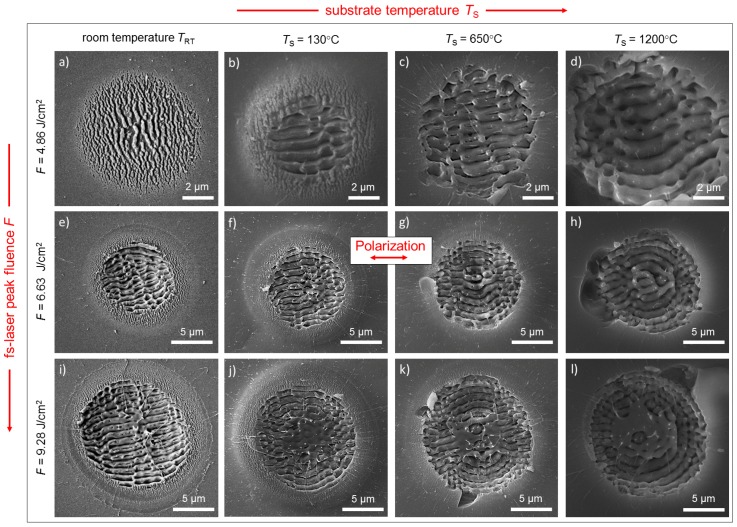
Scanning electron microscopy (SEM) micrographs of the fused silica surface upon irradiation with linearly polarized laser pulses (*N* = 5) at *T*_RT_, *T*_S_ = 130, 650, and 1200 °C using (**a**–**d**) *F* = 4.86 J/cm^2^, (**e**–**h**) *F* = 6.63 J/cm^2^, and (**i**–**l**) *F* = 9.28 J/cm^2^. Note the direction of the beam polarization (red arrow) and the different scaling of the SEM micrographs.

To investigate the influence of the initial substrate temperature, equidistant spots with LIPSS were generated with *N* = 5 linearly polarized laser pulses at a fixed fs-laser peak fluence, *F*. The spots were subsequently characterized according to their position, *r*, at the sample surface, i.e., in dependence on the corresponding initial substrate temperature, *T*_S_(*r*). Figure 3 shows an overview of the SEM micrographs of LIPSS fabricated with different values of *F* (from top to bottom) along with their respective initial substrate temperatures, *T*_S_ (from left to right). At *T*_RT_ as the reference, the lowest value, *F* = 4.86 J/cm^2^, leads to the formation of HSFL with spatial periods, *Λ*_HSFL_, ranging from 200 to 400 nm (0.19·*λ*–0.39·*λ*) (Figure 3a). In this case, the specific value of *Λ*_HSFL_ depends on the respective fluence correlated to the Gaussian intensity distribution of the fs-laser beam. The increase of *F* to 6.63 J/cm^2^ results in the formation of LSFL in the center of the Gaussian beam profile with *Λ*_LSFL_ = (830 ± 115) nm, which are surrounded by a ring-shaped area containing HSFL (Figure 3e). The further increase of *F* to 9.28 J/cm^2^ causes an increase of the diameter, *D*_LSFL_, of the area covered with LSFL, and finally results in an extended pattern containing homogeneously distributed LSFL characterized by *Λ*_LSFL_ = (860 ± 81) nm (Figure 3i). The micrographs at *T*_RT_ demonstrate that the HSFL are aligned perpendicular and the LSFL are parallel to the direction of the E-field vector of the linearly polarized fs-laser radiation. Together with the spatial periods determined at *T*_RT_, this is in good agreement with results already published for fused silica, whereby a direct comparison with the work of Höhm et al. is complicated by deviating wavelengths, pulse numbers, and pulse durations [10,11]. At elevated temperatures, the micrographs in Figure 3 reveal remarkable differences in the size of the area covered with LSFL and the properties of the occurring LIPSS, particularly in terms of their type, morphology, spatial period, and alignment.

#### 3.2.1. Temperature-Dependency of the LIPSS Formation Threshold

**Figure 4:**
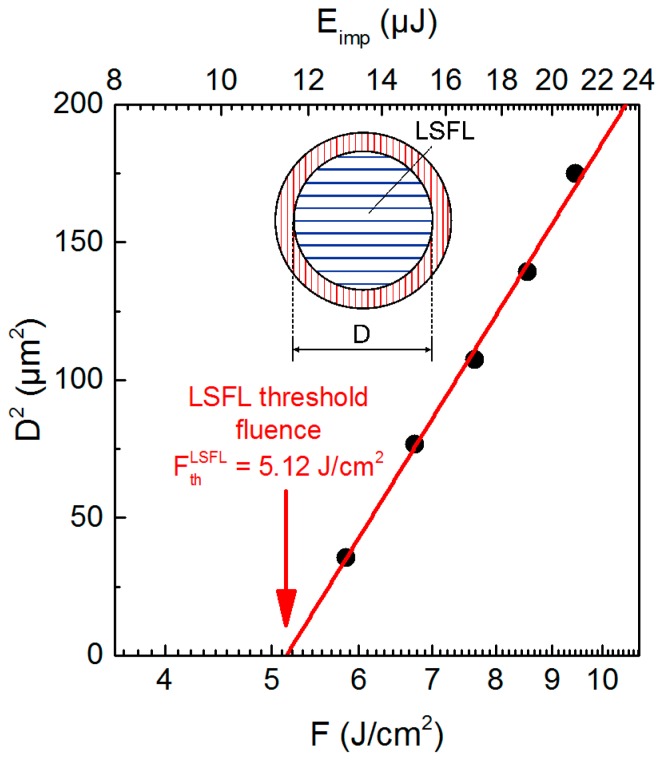
Evaluation of the threshold of low-spatial frequency LIPSS (LSFL) formation upon irradiation with linearly polarized fs-laser pulses (*N* = 5) using the method proposed by Liu [34].

**Figure 5:**
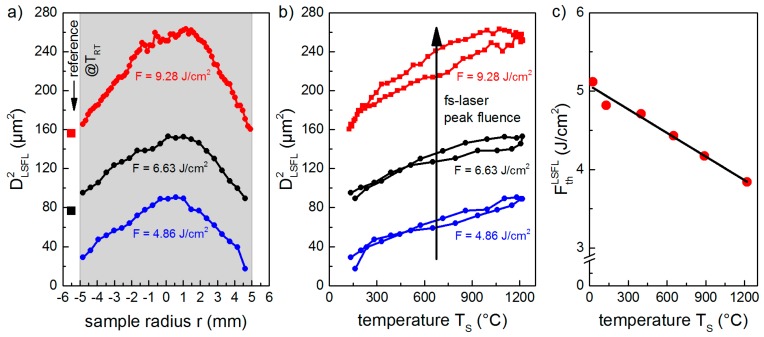
Evaluation of the area covered with LSFL (diameter *D*_LSFL_): (**a**) *D*^2^_LSFL_ in dependence on the sample radius, *r*, (**b**) *D*^2^_LSFL_ in dependence on the corresponding substrate temperature *T*_S_ and (**c**) temperature-dependency of the threshold *F*_th_^LSFL^ for the formation of LSFL. *T*_RT_ indicates room temperature.

The results obtained at *T*_RT_ (Figure 3a,e,f) indicate a transition from HSFL to LSFL at a specific threshold fluence, *F*_th_^LSFL^, which was quantified by plotting *D*^2^_LSFL_ in a semilog plot versus *F*, as proposed by Liu [30] (Figure 4). Extrapolating the linear fit of the measured diameters to zero provides the value of *F*_th_^LSFL^, which was found to be 5.12 J/cm^2^ for the used processing conditions, in particular the pulse number, *N* = 5. To determine the influence of *T*_S_ on the formation process, *D*_LSFL_ was measured in dependence on the position, *r*, at the sample surface (Figure 5). Figure 5a shows that *D*^2^_LSFL_ increases with increasing *F* in the investigated temperature range. In addition, at a fixed *F*, the graph reveals an increase of *D*^2^_LSFL_ towards the center of the sample (*r* = 0), i.e., *D*^2^_LSFL_ also increases with increasing *T*_S_ (Figure 5b). Note that Figure 5b shows small deviations resulting from asymmetries in the temperature profile, which are attributed to the angle of incidence of the CO_2_ laser beam and inhomogeneities in its intensity profile. The determined dependency of *D*_LSFL_ suggests a decrease of the corresponding threshold fluence with increasing *T*_S_, which, similarly, has been reported by Bude et al. for the ablation threshold of fused silica [30]. In contrast, Yahng and co-workers evaluated that, for the micro-processing of glass, both the ablation depth and the ablation threshold are almost invariant to an increase of *T*_S_ [29]. In the present study, the determined decrease of *F*_th_^LSFL^ is confirmed by the comparison of LIPSS fabricated with *F* = 4.86 J/cm^2^ at *T*_RT_ (Figure 3a) and at *T*_S_ = 130 °C (Figure 3b). The micrographs reveal that, although *F* was kept below the room temperature formation threshold, LSFL are generated in the center of the fs-laser spot at the elevated temperature of *T*_S_ = 130 °C.

Moreover, the value measured for *F*_th_^LSFL^ continuously decreases from its initial value of 5.12 J/cm^2^ at *T*_RT_ to 3.84 J/cm^2^ at *T*_S_ = 1200 °C (Figure 5c). As expected, the prior heating of the substrate by the CO_2_ laser leads to larger diameters of the LIPSS pattern for a given laser fluence, *F*, and, therefore, the transition from HSFL to LSFL starts at lower laser peak fluences when compared to *T*_RT_.

#### 3.2.2. Temperature-Dependency of the LIPSS Spatial Period

**Figure 6:**
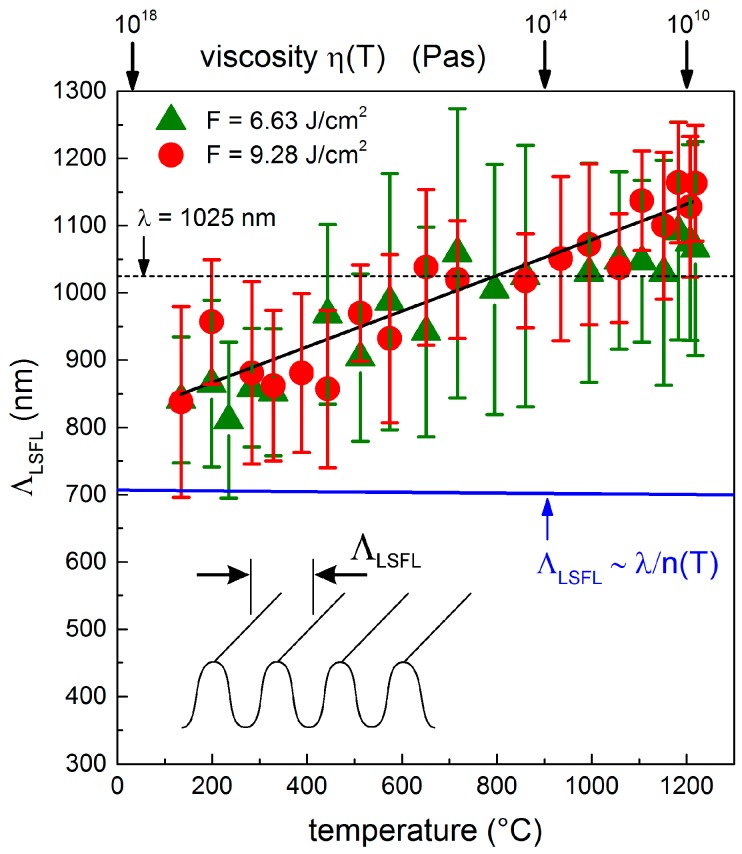
Spatial period, *Λ*_LSFL_, of the LSFL (*N* = 5) as a function of the initial substrate temperature, *T*_S_. The vertical bars indicate the width of distribution of the corresponding 2D-FT operation and the black solid line guides the eye. The blue line corresponds to the spatial periods, *Λ*_LSFL_, predicted by the Sipe theory considering a temperature-dependent refractive index, *n*.

For a given laser peak fluence, *F*, Figure 3 indicates a remarkable increase of *Λ*_LSFL_ with increasing *T*_S_. This dependency was quantified by 2D-FT. The resulting Figure 6 shows that at low surface temperatures, *Λ*_LSFL_ is equal to the spatial periods of the structures generated at *T*_RT_ (*Λ*_LSFL_ = 830–860 nm). Moreover, the results confirm that the increase of *T*_S_ leads to increasing spatial periods. At the maximum investigated temperature of *T*_S_ = 1200 °C, *Λ*_LSFL_ reaches a value of (1170 ± 90) nm for *F* = 9.28 J/cm^2^. Note that for *T*_S_ ≈ 800 °C, *Λ*_LSFL_ is equal to the wavelength, *λ* = 1025 nm, of the fs-laser beam. For *T*_S_ > 800 °C, the observed laser-induced structures correspond to “supra-wavelength” LIPSS. Here, the spatial period exceeds *λ* at the maximum by a factor of about 1.14 at *T*_S_ = 1200 °C, i.e., *Λ*_LSFL_ is increased by about 36% when compared to *T*_RT_. From the experimental results illustrated in Figure 6, a d*Λ*/d*T* slope can be calculated, which was found to be 0.24 nm·K^−1^ for the used processing conditions. It must be noted that the width of the distribution of the corresponding 2D-FT operation (indicated by the vertical bars) is only slightly increased when compared to the spatial periods measured at *T*_RT_.

In addition to the determined increase of *Λ*_LSFL_ with *T*_S_, Figure 3 reveals that HSFL are mainly formed at *T*_RT_ both at laser peak fluences below the formation threshold, *F*_th_^LSFL^ (Figure 3a), and at higher fluences in the vicinity of the LSFL (Figure 3e,i). In contrast, HSFL cannot be observed in the micrographs at elevated temperatures (e.g., *T*_S_ = 1200 °C in Figure 3d). This might be related to the specific mechanism of their formation. It can be found in the literature that the formation of HSFL is associated with a very rapid energy deposition into the material, and thus with a strongly localized energy [20]. In contrast to LIPSS formation at *T*_RT_, this energy localization might be washed out in a heated substrate, which means that HSFL are no longer visible. Considering the viscosity, it can also be argued that the surface softens during CO_2_ laser heating and, therefore, thermal diffusion may lead to a smoothing of the very small HSFL. Moreover, Figure 3 shows, in accordance with the literature, the generation of well-ordered LSFL with an orientation parallel to polarization at lower temperatures [10,11]. At elevated temperatures (e.g., *T*_S_ > 650 °C in Figure 3k,l), however, the LSFL are no longer parallel to the beam polarization. Instead, they exhibit a concentric alignment according to the boundary conditions given by the solid border of the ablation crater. This might be correlated with temperature-induced melt formation and hydrodynamic surface waves. To analyze possible mechanisms, we performed the following estimations using both electromagnetic and hydrodynamic models.

### 3.3. LIPSS Analysis by Sipe Theory

The theoretical analysis of LIPSS formation is most commonly conducted based on the theory of Sipe et al. [9], which was discussed for fused silica and other band gap materials in a series of works [10,11,36,37]. This theory provides an “efficacy factor”, *η*, that determines the coupling efficiency of light at the wave vector, ***k***, absorbed by the laser-irradiated material, assuming a homogenous profile of the dielectric permittivity, ε*, and a random surface roughness of low amplitude, *d* << *λ*. A series of LIPSS wave vectors, ***k*** (***k*** = 2π/*λ*), as a function of the laser parameters (laser wavelength: *λ*, angle of incidence: *θ*, and the polarization direction) and of the materials’ properties (the dielectric permittivity: *ε*, and two surface roughness parameters: Shape factor, *s*, and the filling factor, *f*) can therefore be obtained and compared to LIPSS periodicities measured for a specific material. The complex dielectric permittivity, *ε* = *ε*_r_ + i*ε*_i_, is connected to the optical constants, *n* and *k*, via *ε* = (*n* + i*k*)^2^. For fused silica at *T*_RT_ and *λ* = 1025 nm, *ε* is given by *ε* = (1.4504 + i·0)^2^ = 2.1036 [38]. In laser-irradiated band gap materials, the transient excitation of the electrons in the solid leads to a transient modification of the optical properties as a function of the time and of space [20,21]. A simplified description of the optical properties consists of selecting only the peak intensity, and to account for an effective dielectric permittivity, ε*, describing the transient modification of the optical properties at the materials’ surface [8]. If neglecting the formation of self-trapped excitons, the transient change of the optical properties due to the excitation of electrons over the band gap, *E*_g_ = 9 eV [39], by multi-photon absorption can be approximated by using a Drude model [10,40] expressed by:

(1) ε*=ε+ΔεD=ε−e2·Neε0·mopt·me·ω2(1+iω·τD)

Here, *e* represents the electron charge, *N*_e_ is the laser-excited electron density, *m*_e_ is the electron mass, *ε*_0_ is the vacuum dielectric permittivity, and *ω* the laser angular frequency. Typically, a Drude damping time of *τ*_D_ = 0.4 fs and an optical effective mass of *m*_opt_ = 0.49 are used for the calculations concerning fused silica [41]. The shape factor, *s*, and the filling factor, *f*, are set to 0.4 and 0.1, respectively [9]. Note that the Drude model validity to describe the change of optical properties in SiO_2_ upon ultrafast laser-irradiation is limited to intensities up to 10^14^ W/cm^2^ [42]. The results of this approach for the cold material were presented in previous studies [10,11]. It was shown that the sub-wavelength periods of LSFL observed for fused silica at *T*_RT_ (see also Figure 3e,i) can be explained adequately taking into account the Drude model (Equation (1)). 

**Figure 7:**
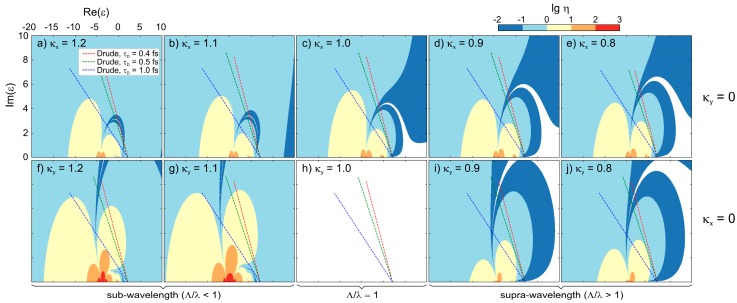
Sipe efficacy factor, *η*, presented as a function of the complex dielectric permittivity, *ε* [Re(*ε*), Im(*ε*)]. For the calculations, polarization was oriented towards the *x*-direction. In the first line (**a**–**e**), *η* was computed for *λ*/*Λ*_y_ = *κ*_y_ = 0 and *κ*_x_ was systematically varied. In the second line (**f**–**j**), *η* was computed for *λ*/*Λ*_x_ = *κ*_x_ = 0 and *κ*_y_ was systematically varied. The white zones indicate an efficacy factor smaller than 10^−2^. The false-color scale is the same for all the sub-figures: The cold color indicates an efficiency factor lower than 1, and the warm color indicates an efficiency factor greater than 1. The dashed lines correspond to the density-dependent excited dielectric permittivity, ε*, of fused silica upon excitation computed using the Drude model (see Equation (1)). The impact of different Drude damping times, *τ*_D_, are represented and the optical mass was kept at *m*_opt_ = 0.49.

In the available literature, Sipe model predictions were performed for a specific material by finding local maxima in *η* as a function of the space of normalized wavenumbers, *κ*, along with *N*_e_ [9,10,11,36,37]. In an alternative approach, we use the Sipe model to identify the effective dielectric permittivity, ε*, at which the sub-wavelength and supra-wavelength periodic deposition of energy could be obtained. For this purpose, *η* was analyzed in the space of complex dielectric permittivities, [Re(*ε*), Im(*ε*)], that can be achieved during the laser irradiation for selected sub- and supra-wavelength wavenumbers. Figure 7a–j presents a collection of Sipe efficacy factors presented in false-color figures with the logarithmic scale calculated for several spatial frequencies in the *x*- and *y*-direction, respectively. Note that the wave-vectors, *κ*, are normalized to unity. The warm colored areas correspond to *η* > 1, whereas cold colored areas indicate *η* < 1. Assuming that the local field amplification scales with *η*, the maps calculated for *κ*_y_ = 1.2 (Figure 7f) and *κ*_y_ = 1.1 (Figure 7g) correspond to the regime of sub-wavelength energy deposition, which is characterized here by *η* > 1 (yellow-colored area). The dashed lines in Figure 7 represent the specific case of fused silica computed with the Drude model with a dependence on *N*_e_ (Equation (1)). Here, different values of *τ*_D_ were used to identify the influence of the Drude damping time at a constant optical mass of *m*_opt_ = 0.49. The calculations confirm that the sub-wavelength regime can be reached transiently during laser irradiation of fused silica, which allows, in accordance with the previous studies [10,11], predictions of the LIPSS periodicities at *T*_RT_.

Conversely, supra-wavelength structures are associated with normalized wavenumbers, *κ*_y_ < 1. The maps calculated for *κ*_y_ = 0.9 (Figure 7i) and *κ*_y_ = 0.8 (Figure 7j) show the principle possibility of supra-wavelength energy deposition according to the Sipe theory. In this case, the most favorable effective dielectric permittivity is located in the maps close to ε* ~ −5 + i. At equilibrium, this region corresponds to metals with optical losses such as Cr, W, and Ti [43]. However, it can also be reached by transiently excited band gap materials. However, the evolution of *η* during laser excitation of the fused silica sample, which is represented by the dashed lines, leads to values of *η* smaller than 1 (bright-blue colored areas). This suggests that the conditions to obtain supra-wavelength energy deposition are not met during the irradiation of SiO_2_, provided that the Drude model would be sufficient to describe its transient optical response. The same argumentation applies in the other direction (*y*-axis) perpendicular to polarization: A supra-wavelength periodic energy deposition remains possible under similar conditions as in the direction of the *x*-axis (corresponding to TM mode), although computed values of *η* are lower in comparison to the efficacy factor found for the sub-wavelength periodicities (*κ* > 1). Note that, according to the Sipe model, periodicities strictly equal to laser wavelengths in the TE mode are not possible (Figure 7h). It has to be emphasized that the pre-heating of the sample by the CO_2_ laser investigated in the present study leads to a negligible change of the refractive index of fused silica (d*n*/d*T* = 1.15∙10^−5^ K^−1^) [38], and thus of the dielectric permittivity. Consequently, no significant modification of *η* and therefore of the dashed lines in Figure 7 occurs, i.e., the regime of supra-wavelength electromagnetic energy deposition will be not be reached by substrate heating (therefore not presented here). The marginal impact of temperature on the Sipe-predicted periods is also confirmed by the blue line in Figure 6. We can thus conclude that the spatial periods obtained from the Sipe model analysis do not match with the experimentally observed increase of the spatial periods with increasing *T*_S_ (Figure 6), although the Sipe model basically allows the explanation of the formation of supra-wavelength periodic structures by an electromagnetic energy deposition phenomena, but only in conditions that are not described by the Drude model.

### 3.4. Hydrodynamic Non-Dimensional Numbers Analysis and Thermo-Capillary Instability

As the effect of melt formation and viscosity appears relevant in contrast to the transient change of the optical properties, a hydrodynamic study was performed, as already suggested in our previous work [11]. To predict the hydrodynamic instabilities that may occur in the laser-induced molten layer after laser irradiation, simple estimations are performed using non-dimensional numbers [44]. They allow indications to be made on the relative importance of forces, energies, and time scales. Although theoretical modeling of surface melting was performed in the past with ns-laser beams [45,46,47], time-resolved pump-probe imaging of laser-irradiated semiconductors [48] revealed an energy-dependent lifetime of the molten material in the range of 10 ns. This makes analytical calculations challenging in the case of fs-laser irradiation. In this context, recent theoretical studies have shown that surface gradients of temperature may be sufficient to enable the development of surface hydrodynamic instabilities on metals and semiconductors [49,50], whereas non-dimensional number analysis has been proposed for laser-irradiated metals [51]. Although recent time-resolved imaging of laser-irradiated semiconductors could not strictly lead to the emergence of grating-like LIPSS after a single pulse [27], such a possibility was observed in specific laser-irradiated metals where LIPSS were seemingly originating from hydrodynamic effects [52]. To shed light on the laser-induced hydrodynamic phenomena under our experimental conditions, in the present study, a similar approach was applied to investigate the possible regimes of molten layer transport in the case of hot fused silica.

Table 1 presents a series of non-dimensional numbers calculated from the Navier-Stokes equation for the superficial layer of hot molten fused silica using the materials’ data given in Table A1. Here, the radius of curvature, *R*, is given by 1/*R* ≃ ∂²*z*/∂*x*² + ∂²*z*/∂*y*^2^ ∼ 2*h*/*L*^2^ where *h* is the molten depth (comprised between 1 and 200 nm) and *L* is the typical size of the system, comprised between 1 and 5 µm. The results of this analysis suggest that the convective transport regime dominates over the diffusive transport of the liquid, and that the free surface of the hot liquid can be rapidly deformed by the temperature-dependent surface tension gradients. These gradients can be induced by the flow of the hot liquid fused silica that it is reaching and therefore deforming the surface during the lifetime of the moving molten layer. These results strongly suggest that a thermo-convective instability, driven by the surface tension modification, may be acting in the molten layer after the laser pulse irradiation.

**Figure 8:**
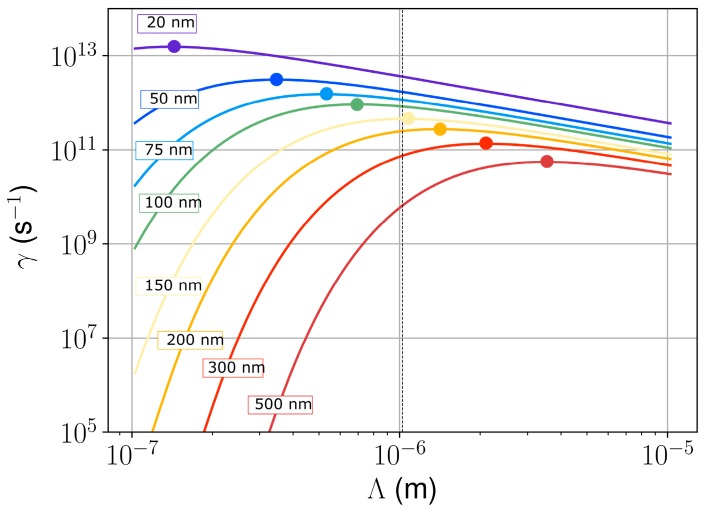
Growth rate, *γ*, of the thermo-convective instability as a function of the period of the unstable mode at *T* = 2000 K. Dots identify the most unstable mode for thin layers of hot fused silica, varying the thickness, *h*, from 20 to 500 nm.

To quantify this effect, a model of thermo-convective transport was used to compute the possible periodicities that can emerge by the development of thermo-capillary instability in the hot molten fused silica layer with a free surface [54]. This model is based on the Navier-Stokes equation for a shallow liquid layer with a temperature gradient oriented towards the depth of the sample. It provides an instability growth rate, *γ*(*h*, *T*) (in s^−1^), as a function of the liquid molten layer thickness, *h*, the material temperature, *T*, the temperature-dependent surface tension, *σ*(*T*), and the temperature-dependent viscosity, *η*(*T*). The quantities used for these calculations are given in Table A1, and the growth rate calculations at *T* = 2000 K are plotted as a function of the mode periodicity in Figure 8.

Figure 8 reveals that the period of the most unstable mode is a supra-wavelength when the thickness of the molten layer exceeds 150 nm. The corresponding order of magnitude for its development time is *γ*^−1^ ~ 2–20 ps. A small molten layer thickness (lower than 100 nm) leads to sub-wavelength periods of the unstable mode. It is evidenced that the period of the fastest developing mode increases with increasing *h*. Note that, at this stage, the comparison of these theoretical results with experimental results presented in Figure 3 and Figure 6 remains difficult due to the high laser fluence, *F*, that was used to perform the experiment. Upon increasing *F*, the effective optical penetration depth in fused silica decreases due to the multiphoton absorption and to the free-carrier heating [40], inducing melting in a layer of a reduced depth along with material removal (as denoted by the splashes in Figure 3). Note that an exhaustive description of the latter remains difficult as it would require modeling of several removal non-equilibrium phenomena [55], such as Coulomb explosion [40], bubble nucleation, and phase explosion [56,57,58]. The role of these effects on LIPSS formation was recently described in [15]. Also, the importance of the ablation plasma radiation to the irradiated target was mentioned in [55]. All these processes were neglected in our present study. In addition, the temperature that is used in the model may change with space and time, and is therefore different from the initial substrate temperature established by the homogeneous laser irradiation. However, it seems plausible that the thickness of the molten fused silica layer increases with increasing initial substrate temperature, which, according to the presented hydrodynamic analysis, correlates with the experimentally determined increase of the spatial periods with *T*_S_ (Figure 6). More advanced numerical modelling [20,21,55] would be necessary to describe the complete interaction considering the non-trivial absorption of light energy with multiphoton excitation of fused silica, avalanche ionization, phase transition, and material melting, along with an adequate description of the molten material dynamics. In the present stage, our results suggest that a hydrodynamic instability of a thermo-convective nature allows an explanation of the supra-wavelength periodic structures observed on the surface of the laser-heated material. This simple model helps to identify an important physical effect that can be included in existing advanced numerical models to describe the physics of the LIPSS formation in hydrodynamic regimes. A fully integrated modelling of the experiment is beyond the scope of this paper and may require further development of existing tools, since the proposed non-linear convective phenomena, which are described here as thermo-convective capillary instability, are, to our understanding, not included in the existing works [33].

## 4. Conclusions

The formation process and the morphological properties of laser-induced periodic surface structures (LIPSS) were investigated on fused silica as a function of the substrate temperature, *T*_S_. It was shown that the threshold fluence for the formation of low-spatial frequency LIPSS (LSFL) is reduced by increasing *T*_S_. Moreover, the results reveal a remarkable increase of the LSFL spatial periods with increasing *T*_S_, and that surface temperatures above 850 °C lead to the formation of supra-wavelength LIPSS. Theoretical analysis of LIPSS formation demonstrated that the electromagnetic scattering theory is not capable of predicting supra-wavelength structures in the case of fused silica. In contrast, hydrodynamic analyses indicated that thermo-convective instabilities are suitable for the development of supra-wavelength structures if the molten layer is thick enough. However, to achieve a predictive modelling, more advanced numerical simulations are required that consider the convective transport phenomena in the hot molten layer that are usually disregarded.

## Figures and Tables

**Table 1:** Non-dimensional number analysis of the hydrodynamic phenomena in the laser-induced molten layer of fused silica. This interpretation was constructed using Ref. [53] with *L*: Typical size of the system, *h*: Molten layer thickness, *τ*: Relaxation time, *v*: Velocity, *ρ*: Volumic mass, *η*: Dynamical viscosity, *σ*: Surface tension, *c*: Heat capacity, *κ*: Thermal conductivity, *p*: Pressure, and *g*: Gravitational acceleration.

Process	Definition	Magnitude	Regime
momentum damping	*τ*_diff_ = *ρ*·*L*^2^·*η*^−1^	10^−15^ s	strong viscosity dissipates the liquid momentum
ambient-pressure-induced deformation of the interface	*v*_p_ = *p*_out_·*L*/*η* *τ*_p_ = *L*/*v*_p_	10^−9^ m/s ∞	negligible
surface tension driven deformation of the interface	*v*_σ_ = 2·*σ*·*h*/(*L*·*η*) *τ*_σ_ = *L*/*v*_σ_	10^−8^ m/s ∞	negligible
non-linear transport induced by recoil pressure	*v*_NL_ = (*p*_out_/*ρ*)^1/2^ *t*_NL_ = *L*/*v*_NL_	2 m/s 500 ns	considerable effect
non-linear transport due to surface tension gradient	*v*_NLSTG_ = (2·*σ*·*h*/(*ρ*·*L*^2^))^1/2^ *t*_NLSTG_ = *L*/*v*_NLSTG_	1–6 m/s 0.2–1 µs	considerable effect
Reynolds number	*Re* = *ρ*·*v*·*L*/*η*	10^−8^	inertial transport is negligible
Capillary number	*Ca* = *η*·*v*/*σ*	10^7^–10^12^	surface tension dominates over materials viscosity, capillary effects must act at the free surface
Reech number	*Ri* = *g*·*L*/*v*^2^	<5·10^−5^	the influence of gravity is negligible
Peclet number	*Pe* = *η*·*c*_l_/*κ*	~10^16^	thermal convection dominates over diffusion
Weber number	*We* = *ρ*·*v*^2^·*L*/*σ*	0.1–0.4	inertial forces and surface tension are competing

## Appendix A

**Table A1:** Thermophysical properties of quartz, fused silica, and liquid silica.

Quantity	Unity	Quartz	Fused Silica	Liquid Silica
Volumic mass *ρ*	kg/m³	2.5·10³	2.2·10³	-
Fusion temperature *T*_m_	K	1970	1873	-
Heat diffusivity *D*	m²/s	8.6·10^−4^	9·10^−5^	-
Thermal conductivity *κ*	W/(m·K)	0.14 [59]	0.014 [59]	-
		*a*_3_*T*³ + *a*_2_*T*² + *a*_1_T + *a*_0_ [38] *a*_3_ = 7.06418·10^−10^, *a*_2_ = −3.96976·10⁻⁶, *a*_1_ = 7.56664·10^−3^, *a*_0_ = 0.63319		
Heat capacity *c*	J/(g·K)	0.74	0.72	-
Surface tension *σ*	N/m	*aT* + *b*, *a* = 1.54·10^−5^, *b* = 0.267 [60]		
Dynamical viscosity *η*	kg/(m∙s)	0.1·exp(10⁴·*a*/*T* + *b*), *a* = 6.2388, *b* = −14.6668 [61]		
